# Impact of Particulate Matter Exposure and Surrounding “Greenness” on Chronic Absenteeism in Massachusetts Public Schools

**DOI:** 10.3390/ijerph14020207

**Published:** 2017-02-20

**Authors:** Piers MacNaughton, Erika Eitland, Itai Kloog, Joel Schwartz, Joseph Allen

**Affiliations:** 1 Department of Environmental Health, Harvard T.H. Chan School of Public Health, Boston, MA 02215, USA; erikaseitland@gmail.com (E.E.); joel@hsph.harvard.edu (J.S.); jgallen@hsph.harvard.edu (J.A.); 2 Department of Geography and Environmental Development, Faculty of Humanities and Social Sciences, Ben-Gurion University of the Negev, Beer Sheva P.O.Box 653, Israel; ikloog@bgu.ac.il

**Keywords:** PM_2.5_, air pollution, NDVI, greenness, absenteeism, public schools

## Abstract

Chronic absenteeism is associated with poorer academic performance and higher attrition in kindergarten to 12th grade (K-12) schools. In prior research, students who were chronically absent generally had fewer employment opportunities and worse health after graduation. We examined the impact that environmental factors surrounding schools have on chronic absenteeism. We estimated the greenness (Normalized Difference Vegetation Index (NDVI)) and fine particulate matter air pollution (PM_2.5_) within 250 m and 1000 m respectively of each public school in Massachusetts during the 2012–2013 academic year using satellite-based data. We modeled chronic absenteeism rates in the same year as a function of PM_2.5_ and NDVI, controlling for race and household income. Among the 1772 public schools in Massachusetts, a 0.15 increase in NDVI during the academic year was associated with a 2.6% (*p* value < 0.0001) reduction in chronic absenteeism rates, and a 1 μg/m^3^ increase in PM_2.5_ during the academic year was associated with a 1.58% (*p* value < 0.0001) increase in chronic absenteeism rates. Based on these percentage changes in chronic absenteeism, a 0.15 increase in NDVI and 1 μg/m^3^ increase in PM_2.5_ correspond to 25,837 fewer students and 15,852 more students chronically absent each year in Massachusetts respectively. These environmental impacts on absenteeism reinforce the need to protect green spaces and reduce air pollution around schools.

## 1. Introduction

For the first time in history, the U.S. Department of Education recently reported chronic absenteeism data for nearly every public school in the country. They found that for the 2013–2014 academic year, over 6.5 million students (kindergarten to 12th grade, ages 5 to 18) missed at least 15 days of school or 10% of the school year for any reason [1]. This trend is exemplified nationwide: 10% of Tennessee students in kindergarten to 3rd grade [2], 15 percent of Mississippi public school students [3], and 20% of all students in Oregon were chronically absent during the 2013–2014 school year [4]. It is essential we identify all potential drivers of chronic absenteeism to ensure the long-term success of students across the United States.

Missing a total of three or more weeks of school due to unexcused or excused, consecutive or non-consecutive absences can lead to low academic achievement, in terms of both attrition and test performance [5]. Absenteeism in earlier grades can result in a reduction of math and reading scores and an increased likelihood of dropping out [6,7]. Even missing three or more days of school resulted in lower National Assessment for Education Progress scores, compared to students with fewer than three absences [8]. In primary and secondary school students, chronic absenteeism serves as an important health performance indicator (HPI) [9], given that absences are often due to health reasons or circumstances beyond their control (i.e., asthma, transportation, unstable housing) instead of oppositional or defiant behavior that would cause them to drop out [6]. For instance, the latest Center for Disease Control estimates revealed that childhood asthma leads to 13.8 million missed school days each year and is the leading cause for absenteeism [10]. Chronic absenteeism can affect success later in life, including impacts on education attainment, employment opportunities, incarceration risk, health status, and financial stability [5,7].

On average, students spend 6.64 hours in school a day for 180 days a year, which translates to nearly 1200 h each year [11]. Therefore, schools have the potential to play a significant positive or negative role on health and academic performance, and this influence extends beyond internal factors. Access to surrounding greenness of a school is positively associated with academic performance, test scores [12], restored attention capacity, decreased stress levels [13], cognitive development [14], reduced mental fatigue and aggression, and improved coping with Attention Deficit Disorder [15]. In schools with more play areas, students have higher levels of physical activity [16]. Green space provides opportunities for physical activity, and may improve cognitive function, learning, and memory through exercise [17,18,19] or through other mechanisms; time in nature can foster children’s imagination and creativity, cognitive and intellectual development, and social relationships [20,21].

Conversely, poor air quality has a well-established negative association with mortality risk, cardiovascular health risks, reduced lung function, adverse birth outcomes, and an exacerbation of preexisting conditions (i.e., asthma) [22,23,24,25,26]. Traffic-related air pollution is associated with higher prevalence and incidence of asthma, which disproportionately impacts urban students [27]. Children are particularly vulnerable to particulate matter with an average diameter less than 2.5 microns (PM_2.5_) exposure because they have greater lung epithelial layer permeability, developing immune systems, larger lung surface areas, and breathe 50% more air per kilogram of body weight compared to adults [28].

Beyond diminished lung function, studies have shown that children exposed to air pollution have poorer cognitive functioning, impaired neurological function, and lower intelligence quotient (IQ) scores compared to non-exposed children [29,30]. Children’s neuropsychological development can be negatively impacted by exposure to nitrogen dioxide, PM_2.5_, and polycyclic aromatic hydrocarbons [31] and exposure to traffic-related air pollutants contributes to impaired brain development among children [32]. Air pollution levels around schools have been linked to poorer student performance [30], lower individual student grade point averages [33], and reductions in sustained attention [34].

We sought to evaluate the impact of two environmental factors—green space and air pollution—on chronic absenteeism in schools. We evaluated PM_2.5_ concentrations and green space around schools in Massachusetts, where more than 950,000 students are enrolled in public and charter schools each year. These exposures were compared to chronic absenteeism rates across K-12 public schools during the 2012–2013 academic year. This analysis concluded that improvements in green space and air pollution surrounding schools in Massachusetts may result in reductions of chronically absent students, when controlled for other social factors.

## 2. Materials and Methods

In the 2012–2013 academic year, Massachusetts was home to 1884 public schools and served nearly 1 million students [35]. For the purpose of this analysis, we aggregated school-specific data during the 2012–2013 academic year from several sources: absenteeism information from the Massachusetts Department of Elementary and Secondary Education (ESE), Normalized Difference Vegetation Index (NDVI) and PM_2.5_ data from satellite imaging, as well as descriptive information about the schools from the National Center for Education Statistics (NCES). This information was integrated into a geospatial schools database, Massachusetts’ School Metrics And Research Tool (MA SMART) that contains academic, social, environmental, and demographic information for all schools in Massachusetts.

The U.S. Department of Education defines chronic absenteeism as the students who miss 10% or more of school days in a school year for any reason, which is equivalent to 18 missed days of school [36]. For all elementary and secondary public schools that reported absenteeism rates, we obtained the percent of students classified as chronically absent during the 2012–2013 academic year from the Massachusetts Department of Education [37]. For the 2012–2013 academic year, 112 schools did not report absenteeism rates.

NDVI and PM_2.5_ are derived from satellite-based data. NDVI is measured by NASA’s (National Aeronautics and Space Administration) Moderate Resolution Imaging Spectroradiometer (MODIS) system [38]. MODIS provides global imaging of vegetation conditions every 16 days. The finest resolution images were used in this analysis, which have a pixel size of 250 m^2^. The NDVI values from 1 September 2012 to 10 June 2013 (a total of 16 imaging periods) were averaged to give a general indication of NDVI over the course of the school year, which is higher in the fall and spring and lower in the winter. NASA produces bidirectional images as a quality control measure and provides an indication of the quality of each image. Images that were obscured by clouds, cloud shadows, or heavy aerosols were not included in the average NDVI calculations. As the image provides NDVI with a spatial resolution of 250 m, each school was assigned an NDVI from the pixel in which they reside, which reflects the surrounding greenness of the school itself, and not the catchment area for the school.

The MODIS system also provides daily images of aerosol optical depth (AOD), which is a measure of the opaqueness of the atmosphere [39]. AOD indicates the degree of light scattering caused by aerosols in the atmosphere. MODIS uses several different bands to filter out cloud aerosols and isolate the effect of near-ground particles. Previously, Kloog et al. [40] developed hybrid regression models that combine monitored PM_2.5_ concentrations with land use, meteorology, and daily AOD measurements on a 1 km grid. The result is daily PM_2.5_ concentrations in Massachusetts with a spatial resolution of 1 km with mean out-of-sample R^2^ exceeding 0.88. For a more in depth methodology refer to Kloog et al., 2014 [40]. PM_2.5_ concentrations from the nearest point to the school were averaged from 1 September 2012 to 30 June 2013.

NCES produces annual data reports on all public schools in the U.S. [41]. For each school in Massachusetts, we obtained student body information such as enrollment by race and location data as both geographic coordinates and mailing addresses. The enrollment by race was used to calculate the percentage of the student body that classified themselves as white or Caucasian. Median household income from the American Community Survey conducted by the U.S. Census Bureau in 2013 was merged to this dataset based on the residing county of the school. County level data was used because census tract data was not available for 2012 or 2013 and school catchment areas often extend beyond the census tract in which the school is located. Due to the high average household income in Massachusetts, schools were stratified into two categories, high income (mean annual income above $67,500) and low income (mean annual income below $67,500).

### Data Analysis

Generalized linear models were used to test the associations between NDVI, PM_2.5_, and absenteeism controlling for socioeconomic factors such as race and income. Analyses were performed using the open-source statistical package R version 3.0.0 (R Project for Statistical Computing, Vienna, Austria). As chronic absenteeism is a rate, a Poisson link function was applied with the following model specification:

$$Absenteeism_{i}=\beta_{1}+\beta_{2}{\left(Percent\;White\right)}_{i}+\beta_{3}{\left(Income\right)}_{i}+\beta_{4}{\left(NDVI\right)}_{i}+\beta_{5}{\left(PM_{2.5}\right)}_{i}+\beta_{5}{\left(NDVI\times PM_{2.5}\right)}_{i}+\varepsilon_{i}$$

- *Absenteeism* = percent of students chronically absent at school *i*.
- *Percent White* = percent of students who classified themselves as White or Caucasian at school *i*.
- *Income* = 1 if average annual household income in the county in which school *i* resides is greater than $67,500, otherwise 0.
- *NDVI* = average NDVI from 1 September 2012 to 10 June 2013 at school *i*.
- *PM*_2.5_ = average PM_2.5_ concentration (μg/m^3^) from 1 September 2012 to 30 June 2013 at school *i*.
- *NDVI* × *PM*_2.5_ = interaction between NDVI and PM_2.5_.
- *ε* = vector of errors between schools.
- *i* = 1,…, 1772 public schools with absenteeism data in Massachusetts.

## 3. Results

The average NDVI for all schools across Massachusetts of 0.5 corresponds to a near infrared reflectance (NIR) of 0.45 and a visible red reflectance of 0.15, which is typical of a temperate climate. As expected, NDVIs were lower during the winter months and lower than annual averages since summer months were excluded from the calculation. The average PM_2.5_ concentration during the academic year around the schools in this analysis of 7.22 μg/m^3^ (standard deviation (SD) = 0.95 μg/m^3^) is below the National Ambient Air Quality Standard of 12 μg/m^3^. All schools were below this level during the 2012–2013 academic year with the exception of 10 schools located in the vicinity of Springfield, Massachusetts. PM_2.5_ concentrations are partly driven by emissions from vehicle exhaust. The average number of miles driven a year by vehicles within 100 m of each the school was derived from the Massachusetts Department of Transportation traffic count data. For every additional million miles driven a year, PM_2.5_ concentrations increased 0.11 μg/m^3^ (*p* value < 0.0001) in the vicinity of the school.

**Figure 1:**
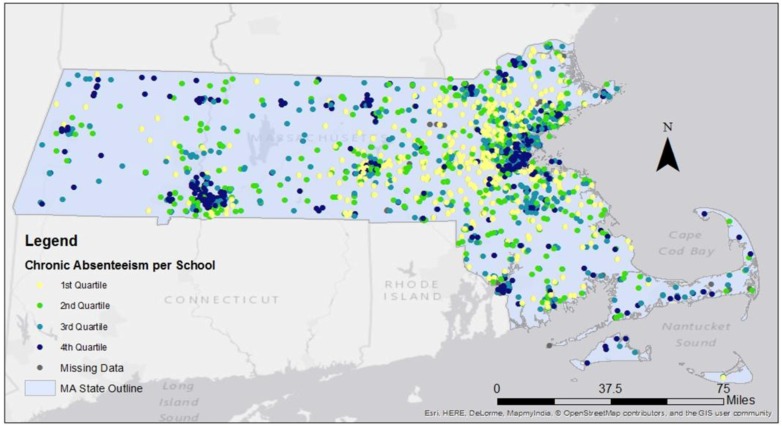
Quartiles of chronic absenteeism per school. Each point on the map is a school (*n* = 1772). 1st quartile (0.0–5.5), 2nd quartile (5.5001–9.30), 3rd quartile (9.3001–15.30), and 4th quartile (15.30–100). MA: Massachusetts.

Figure 1 visualizes chronic absenteeism quartiles by school thus providing a geographical representation of the findings in Table 1. Schools with the highest chronic absenteeism were near urban centers such as Boston, Worcester, Fall River, and Springfield (MA). In Massachusetts, almost a third of schools have higher chronic absenteeism than the national average (13%), despite an average daily attendance of 92% amongst those schools. Schools that had chronic absenteeism rates lower than the national average were on average more white (76.6% white students) than schools that exceeded the national average (47.5% white students).

**Figure 2:**
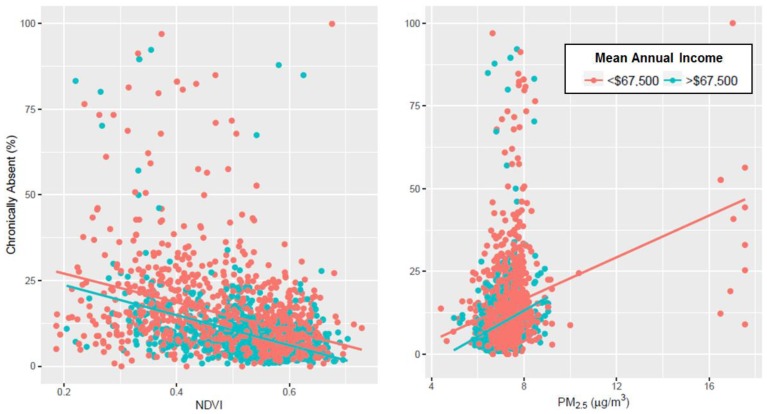
Relationship between NDVI and PM_2.5_ on the percent of students chronically absent during the 2012–2013 academic year at each school in Massachusetts (USA), stratified by counties with a mean annual income above and below $67,500.

The means and distributions of these variables were calculated for schools in both low income and high-income counties (Table 1). The schools in high-income counties had less variability in environmental and social contextual factors. The standard deviations of NDVI, PM_2.5_, percentage of white students, annual household income, and chronic absenteeism were smaller in the high-income counties, indicating that student populations and siting in these counties are more similar to each other than those in the low-income counties, which have a diverse set of environmental and social contexts. Figure 2 shows the relationship between NDVI, PM_2.5_, and chronic absenteeism, stratifying by schools in counties with average annual household incomes above and below $67,500. The elevated PM_2.5_ levels at schools around Springfield did not significantly affect the slope of this relationship when excluded. In addition, the relationships are linear within the range we observed, indicating that there is not a clear threshold of adequate PM_2.5_ or NDVI.

The relationships between NDVI, PM_2.5_ and chronic absenteeism were modeled using generalized linear models to control for the social contextual variables and test for effect modification between social and environmental variables (Table 2). An interquartile range (IQR) increase in NDVI was associated with a 2.6% (*p* value < 0.0001) reduction in the number of students chronically absent, and an IQR increase in PM_2.5_ was associated with a 1.15% (*p* value < 0.0001) increase in absenteeism. The interaction term shows an even larger effect of PM_2.5_ when NDVI is high and a dampening in the effect of PM_2.5_ when NDVI is low. The social contextual variables (percentage of white students and county-level household income as a dichotomous variable) were also significantly associated with absenteeism. The R^2^ for this model indicates that 23.3% of the variability in absenteeism rates is explained by these model parameters.

## 4. Discussion

In our study of Massachusetts schools, surrounding greenness and ambient particulate matter were significantly associated with chronic absenteeism, even when race and income are accounted for: an IQR increase in NDVI was associated with a 2.6% lower chronic absenteeism rate, and an IQR increase in PM_2.5_ was associated 1.15% increase in chronic absenteeism rates. The baseline prevalence of chronically absent students in the Massachusetts public schools in 2012–2013 is 12.6%, so these percentages reflect a significant proportion of the total chronic absenteeism cases observed. In Figure 2, schools, in counties with high household incomes, are associated with high NDVI/low absenteeism and low PM_2.5_/low absenteeism regions of the charts. Regardless of the income bracket in which the school is located, the relationship between NDVI and absenteeism was found to be positive and the relationship between PM_2.5_ and absenteeism to be negative. The effects of surrounding greenness and air pollution do not appear to be entirely additive, suggesting that surrounding greenness and air pollution can independently affect chronic absenteeism rates even if the other factor is adequate. When subsetted to students of different ages, these effect estimates became more pronounced in high schools (9th grade to 12th grade) than elementary schools (prekindergarten to 8th grade). As students get more autonomy in attending school, not only do absenteeism rates tend to be higher but they also become more likely to miss school due to their environmental context.

The variability in NDVI and PM_2.5_ across Massachusetts is small, leading to IQRs of 0.15 for NDVI and less than 1 μg/m^3^ for PM_2.5_. Based on the result from the model, shifting the exposure to NDVI and PM_2.5_ by 0.15 or 1 μg/m^3^ would prevent 25,837 students and 15,852 students respectively from being chronically absent, holding other confounders constant. These estimates are derived by multiplying the predicted percentage changes in chronic absenteeism rates by the total population of students in public schools in Massachusetts. In regions with larger disparities in environmental exposures, a greater percentage of absenteeism rates would likely be attributable to these exposures.

PM_2.5_ has been shown to cause health effects that can result in increased absenteeism. An IQR increase in PM_2.5_ in Hong Kong, equivalent to 20.6 μg/m^3^, was associated with a 3.24% increase in the hospital admission rate for asthma among children below the age of 18 [42]. A review of 37 papers reaffirms the association between PM_2.5_ and hospital admission and derives an overall relative risk of 1.023 [43]. The increase in asthma cases and asthma attacks result in more chronic absenteeism: in 2008, 14.4 million lost days of school were attributable to asthma in the U.S. [44].

Surrounding greenness has also been linked to aspects of health such as improved recovery from illness and improved cardiovascular health [45,46]. A study of twins, which controls for the genetic component of health, found that the members of each twin pair who were exposed to higher NDVI levels had lower depression rates [47]. Access to nature can also promote health behaviors, such as exercise and healthy social interactions [48]. Lastly, research on the same schools as our current study found that NDVI impacts student performance on standardized test scores [12], and it is conceivable that absenteeism may be driving poor performance on standardized tests. We hypothesize that surrounding greenness can lower absenteeism rates by (a) improving students’ hedonic state through improved academic performance and social interaction and (b) improving students’ well-being by promoting exercise, decreasing recovery times from illness, and reducing depression.

In this analysis, 23.3% of the variability in absenteeism rates is explained by NDVI, PM_2.5_, race, and household income. The attributable risk of environmental and social context would be even greater with higher resolution and more precise measures of the exposures and with other contextual variables included, such as hedonic state, household educational attainment, other air pollutants, and baseline health characteristics. The satellite-derived exposures are proxies of the actual exposures, and may lead to misclassification. Better characterization of these variables will reduce residual confounding. Figure 2 shows how environmental context is correlated with income, suggesting the importance of including both social and environmental variables in analysis.

Pre-existing schools have little control over their surrounding context, but there are several ways to address extramural issues with intramural solutions. In the case of particulate matter air pollution, schools that are mechanically ventilated can improve the filtration of outdoor air entering their facilities or install standalone filtration units. Better filtration allows for more particles to be removed but in older building other less energy intensive strategies may be required. Improvements to the building envelope can also reduce or eliminate particle intrusion. If school occupants are aware of the surrounding environmental quality, behavior changes such as limited window opening can reduce exposure to poor outdoor air quality. Schools in urban areas can counteract the effect of low levels of surrounding greenness by investing in landscaping and improving access to parks and nature. Implementing structural soil, a medium that supports root growth in urban contexts, can help improve the quality of planted trees when there is limited space [49]. Policies can be adopted to ensure new schools are sited away from major roadways and industrial sources in areas with more access to nature. The racial and economic disparities can be ameliorated at the school district or state level by a commitment to increase diversity (in both primarily minority and primarily majority schools) and balance investment.

The methods used in this study made it possible to begin to characterize the environmental and social context of every school in Massachusetts. NDVI and PM_2.5_ are objective measures that can be uniformly collected for all schools across the United States and the combined effect of social and environmental factors can be analyzed in this multivariate approach. This information has the potential to enhance current strategies for addressing chronic absenteeism by integrating the environmental context.

While PM_2.5_ and NDVI can be assessed every day and every two weeks respectively, absenteeism rates are only reported annually, preventing within-school or within-student analyses of absenteeism in response to changing environmental exposures such as seasonal changes in NDVI and PM_2.5_. Since each parameter is assessed at the school level, we cannot match exposures and chronic absenteeism at an individual level. The satellite methodology used for both these variables is imperfect, and the limitations of MODIS are provided by NASA [39]. NDVI is a proxy for access to green space; it does not actually assess the extent to which students can access and use the surrounding greenness, if present. It also does not account for other non-green recreational spaces such as access to water sources or gyms. Given the narrow range of NDVI and PM_2.5_ exposures in Massachusetts, the results may not be directly applicable to other regions with larger disparities. The relationships are linear within the range of exposures we observed, but may not continue to be linear outside that range. The variables analyzed are not an exhaustive list of social and environmental context variables, but these associations demonstrate several of the pathways by which contextual variables can influence absenteeism rates. Recognizing the limitations of the analysis, future research directions include examining this relationship over time, understanding if there are statistically significant spatial relationships and clustering, and exploring this relationship in a global context with greater diversity of NDVI and PM_2.5_ exposures.

## 5. Conclusions

Ambient air pollution and surrounding greenness are both associated with chronic absenteeism in public schools in Massachusetts. Schools with higher PM_2.5_ levels have higher absenteeism rates, and schools with more surrounding greenness have lower absenteeism rates. The effect sizes of these environmental exposures are nearly as large as those for income and race, which have been extensively studied, indicating that environmental context plays an important role in student attendance. Importantly, schools in low socioeconomic status (SES) areas also tend to have high air pollution and low NDVI scores, compounding the burden of chronic absenteeism in these areas. Addressing these environmental predictors of absenteeism may help reduce the burden on disadvantaged communities. This research provides evidence that schools districts should evaluate their surrounding environmental context by assessing air quality, proximity to roadways and waste sites, access to green space, and other environmental factors. This assessment ultimately promotes community well-being and identifies alternative cost effective methods for reducing chronic absenteeism.

## Figures and Tables

**Table 1:** Descriptive statistics of exposures and outcomes, stratified by average annual income in each school’s county.

Variable	Mean	Standard Deviation	25th Percentile	Median	75th Percentile	IQR	*n*
Total							
NDVI	0.51	0.10	0.44	0.53	0.58	0.14	1876
PM_2.5_ (μg/m^3^)	7.22	0.95	6.80	7.15	7.54	0.74	1877
Percent White	67.3	29.5	50.0	79.9	90.3	40.3	1860
Annual Income ($)	67,700	12,400	55,300	67,300	82,100	26,800	1877
Chronically Absent (%)	12.6	12.3	5.50	9.25	15.1	9.60	1772
Average annual income less than $67,500							
NDVI	0.50	0.11	0.42	0.52	0.58	0.17	1156
PM_2.5_ (μg/m^3^)	7.33	1.13	6.85	7.24	7.63	0.79	1156
Percent White	63.4	33.0	32.6	79.5	90.1	57.5	1142
Annual Income ($)	59,000	6880	53,500	60,500	65,200	11,700	1156
Chronically Absent (%)	14.5	12.8	6.80	10.8	17.7	10.9	1087
Average annual income greater than $67,500							
NDVI	0.53	0.09	0.49	0.54	0.59	0.10	720
PM_2.5_ (μg/m^3^)	7.05	0.54	6.72	7.03	7.35	0.63	721
Percent White	73.5	21.4	63.3	80.0	90.8	27.5	718
Annual Income ($)	81,500	3300	82,100	82,100	84,900	2830	721
Chronically Absent (%)	9.46	10.6	4.30	6.80	11.2	6.90	685

IQR: interquartile range; NDVI: Normalized Difference Vegetation Index; PM_2.5_: fine particulate matter air pollution.

**Table 2:** Generalized linear model of NDVI, PM_2.5_ and percent of students chronically absent in public schools in Massachusetts (USA) during the 2012–2013 academic year, controlling for race and testing for the interaction between NDVI and PM_2.5_. The effect size across the interquartile range (IQR) is shown for each continuous variable.

Parameter	*β*	Std. Error	*p* Value	exp(*β*)	Effect Size Across IQR (%)
Intercept	3.037	0.020	<0.0001	20.1	NA
Percent White	−0.007	0.0003	<0.0001	0.993	−6.05
Income	−0.245	0.015	<0.0001	0.783	NA
NDVI	−1.684	0.085	<0.0001	0.186	−2.57
PM_2.5_	0.073	0.005	<0.0001	1.08	1.15
NDVI × PM_2.5_	0.656	0.054	<0.0001	1.93	1.02

*β*: effect estimate, exp(*β*): exponentiated effect estimate.
